# The role of cancer-associated fibroblasts in breast cancer metastasis

**DOI:** 10.3389/fonc.2023.1194835

**Published:** 2023-07-11

**Authors:** Yi Li, Changyuan Wang, Ting Huang, Xijie Yu, Bole Tian

**Affiliations:** ^1^ Department of Breast Surgery, Sichuan Provincial People’s Hospital, University of Electronic Science and Technology of China, Chengdu, China; ^2^ Department of Pancreatic Surgery, West China Hospital, Sichuan University, Chengdu, China; ^3^ Hepatobiliary Surgery Department II, Guizhou Provincial People’s Hospital, Guiyang, China; ^4^ Department of Endocrinology and Metabolism, Laboratory of Endocrinology and Metabolism, National Clinical Research Center for Geriatrics, West China Hospital, Sichuan University, Chengdu, China

**Keywords:** breast cancer, metastasis, cancer-associated fibroblasts (CAFs), tumor microenvironment (TME), extracellular matrix (ECM)

## Abstract

Breast cancer deaths are primarily caused by metastasis. There are several treatment options that can be used to treat breast cancer. There are, however, a limited number of treatments that can either prevent or inhibit the spread of breast tumor metastases. Thus, novel therapeutic strategies are needed. Studies have increasingly focused on the importance of the tumor microenvironment (TME) in metastasis of breast cancer. As the most abundant cells in the TME, cancer-associated fibroblasts (CAFs) play important roles in cancer pathogenesis. They can remodel the structure of the extracellular matrix (ECM) and engage in crosstalk with cancer cells or other stroma cells by secreting growth factors, cytokines, and chemokines, as well as components of the ECM, which assist the tumor cells to invade through the TME and cause distant metastasis. Clinically, CAFs not only foster the initiation, growth, angiogenesis, invasion, and metastasis of breast cancer but also serve as biomarkers for diagnosis, therapy, and prediction of prognosis. In this review, we summarize the biological characteristics and subtypes of CAFs and their functions in breast cancer metastasis, focusing on their important roles in the diagnosis, prognosis, and treatment of breast cancer. Recent studies suggest that CAFs are vital partners of breast cancer cells that assist metastasis and may represent ideal targets for prevention and treatment of breast cancer metastasis.

## 1 Introduction

According to the cancer statistics released by the World Health Organization in 2020, breast cancer surpassed lung cancer to become the disease with the highest incidence worldwide and the leading cause of cancer-related deaths in women (1). It is a heterogeneous disease with several known subtypes, which can be classified as luminal (hormone receptor positive), HER2 overexpressing, and triple negative. These types exhibit different biological and molecular features, leading to a requirement for different treatment modalities, as well as different response patterns and characteristic differences across clinical outcomes (2). Breast cancer deaths are primarily caused by metastasis, which accounts for 90% of all cancer-associated deaths. The 5-year survival rate for patients with localized breast cancer is 99%, but for those with metastatic breast cancer it is 28% (3). The most common sites of breast cancer metastasis are bone, liver, lung, and brain, among which bone is the most common site of breast cancer metastasis, with 70% of metastatic breast cancers involving bone metastasis (4). To suppress this fatal biological behavior, many researchers have investigated its mechanisms and attempted to identify more molecular targets and relevant treatment approaches.

Tumor metastasis relies on multiple steps. Tumor cells first grow at the primary site, invade the ECM and then the systemic circulation, extravasate into the target organ, and finally grow at the metastatic site (5). Steven Paget (6) proposed the “seed and soil” theory, where the “soil” is the tumor microenvironment (TME). Cancer cells received more attention in earlier studies (7), but recently there has been an increased focus on the importance of TME in breast cancer metastasis (8–10). The development of cancer, including metastasis, depends not only on the tumor cells themselves but also, significantly, on the TME (11, 12). The dynamic TME of the primary tumor is involved in the development and invasion of tumor cells, whereas the metastatic TME play a role in the colonization and growth of tumor cells (13).

The TME consists of numerous components, including stromal cells and non-cellular components, with complex interactions between the tumor and stroma. In breast TME, cellular components include cancer-associated fibroblasts (CAFs), immune cells, inflammatory cells, endothelial cells, pericytes, adipocytes, and bone-marrow-derived cells (14–16). The non-cellular components include soluble factors such as chemokines, cytokines, growth factors, and metalloproteinases (MMPs), as well as insoluble factors such as exosomes and extracellular matrix (ECM) (17). CAFs are the most abundant cells in the TME and have important roles in cancer pathogenesis. They can remodel the structure of the ECM and engage in crosstalk with cancer cells or other stroma cells by secreting growth factors, cytokines, and chemokines, which assist the tumor cells to invade through the TME and achieve distant metastasis (18). Clinically, CAFs not only foster the initiation, growth, angiogenesis, invasion, and metastasis of breast cancer but also serve as biomarkers for diagnosis, therapy, and prognostic prediction (19).

Given the importance of CAFs in breast cancer, we undertook this review to discuss current information about the origins, biomarkers, and subtypes of CAFs, as well as their functions, mainly in breast cancer. We focus particularly on their contributions to breast tumor metastasis and the possible implications for cancer therapy.

## 2 Biological characteristics of CAFs

### 2.1 Biological features

CAFs are special fibroblasts in the stroma surrounding the tumor mass. They are also known as activated fibroblasts, myofibroblasts, peri-tumoral fibroblasts, reactive fibroblasts, or tumor-associated fibroblasts (TAFs) (20).They produce ECM components (including collagens, elastin, proteoglycans, glycosaminoglycans and glycoproteins) (21), hormones, cytokines, proteases, and growth factors. Early in 1971, Gabbiani et al. first reported that myofibroblasts could be seen in granulation tissue during wound healing (22). Then, in 1986, Dvorak proposed the concept of cancer as a wound that does not heal (23). Therefore, myofibroblasts exist not only in wounds but also in the stroma of malignant tumors (24). CAFs or activated fibroblasts, also called myofibroblasts, acquire higher abilities of proliferation and migration compared with normal fibroblasts (NFs) (25).

### 2.2 Biomarkers

Various proteins have been reported to show higher expression in CAFs, including alpha smooth muscle actin (α-SMA) (26), tenascin-C (27), chondroitin sulfate proteoglycan (NG2) (28), fibroblast-specific protein (FSP)-1/S100A4 (28), platelet-derived growth factor receptors (PDGFR)α/β (29), fibroblast activation protein (FAP) (30), and podoplanin (31). These are classic biomarkers of CAFs because of their wide application. Different molecular markers, however, have been identified in different CAF subtypes, demonstrating that CAFs represent heterogeneous populations of cells with distinct roles in regulating cancer progression and metastasis (28). Moreover, the expression of these markers in CAFs varies at different metastasis sites. Kim et al. analyzed 132 specimens of breast cancer metastases by immunohistochemistry and found that the expression of CAF-related proteins in the stroma varies with the location of breast cancer metastasis: in lung metastasis, PDGFRα is highly expressed; in liver metastasis, S100A4 and PDGFRα have low expression; and in bone metastasis, podoplanin, S100A4, and PDGFRα are highly expressed (32). Using immunofluorescence, Jaroslaw S. et al. observed that PDPN-positive CAFs colocalized with blood vessels stained with anti-CD34 antibodies in tumor stroma of IDC patients (33). It was Yamazaki and Eyden who first described CD34+ fibroblasts in mammary stroma and intralobular fibroblasts in the breast. But the majority of tumor stroma in ductal carcinoma in situ(DCIS) and invasive breast cancer of no special type (IBC-NST) were characterized by α-SMA positive myofibroblasts rather than CD34+ fibroblasts, several CD34+ fibroblasts are preserved in invasive lobular carcinomas (ILC) (34). In mouse triple-negative breast cancer (TNBC), multiple CAF subpopulations coexist, and the abundance and dynamics of each marker depend on the tumor type and time (35).

In addition to these classic markers, many other markers have been identified. Recently, studies have reported other molecules with higher expression in breast CAFs, including FGF2 (36) and EZH2 (37). One study used bioinformatics analysis to identify a CAF subtype based on gene expression profiles (COL10A1, COL11A1, CXCL11, CXCR6, ADAMTS12, AEBP1, EDNRA, EPPK1, and WNT7B), which was associated with significantly different overall survival rates, proportions of immune cells, and immunotherapy response rates in TNBC (38). In both mouse models and in patients, interleukin (IL)-33 is upregulated in fibroblasts associated with breast cancer metastases, especially lung metastases (39). IL-33 can activate type 2 inflammation in the metastatic microenvironment and facilitate enrollment of eosinophils, neutrophils, and inflammatory monocytes to metastasis sites (39). Integrin α11 is a cell-surface receptor that binds to collagen and other ECM molecules. When it is expressed in CAFs, it helps them to remodel collagen in the TME, allowing them to migrate and contribute to tumor progression (40). CAFs and breast cancer patients’ stroma express high levels of PYCR1, which plays a key part in proline synthesis. *In vivo* and *in vitro*, reducing PYCR1 levels in CAFs reduces tumor collagen production, tumor growth, and metastatic spread (41).

### 2.3 Cellular origins and activation pathways

CAFs comprise a complex and heterogeneous group of cells (42). Their characteristics and molecular markers differ, possibly because of their different cellular origins, which are presumed to form six dominant categories. The majority are NFs, followed by human mesenchymal stem cells (hMSCs) (43–46), and other transdifferentiated cells including endothelial cells, epithelial cells, adipocytes, and pericytes (47–52). Especially in invasive lobular carcinoma of the breast, resident CD34+stromal cells/Telocytes provide a significant proportion of CAFs (53). Compared with these cells of different origin, the process of converting CAF deserves more attention. Multiple pathways have been reported, predominantly involving induction by tumor cells.

#### 2.3.1 Induction by tumor cells

**Figure 1:**
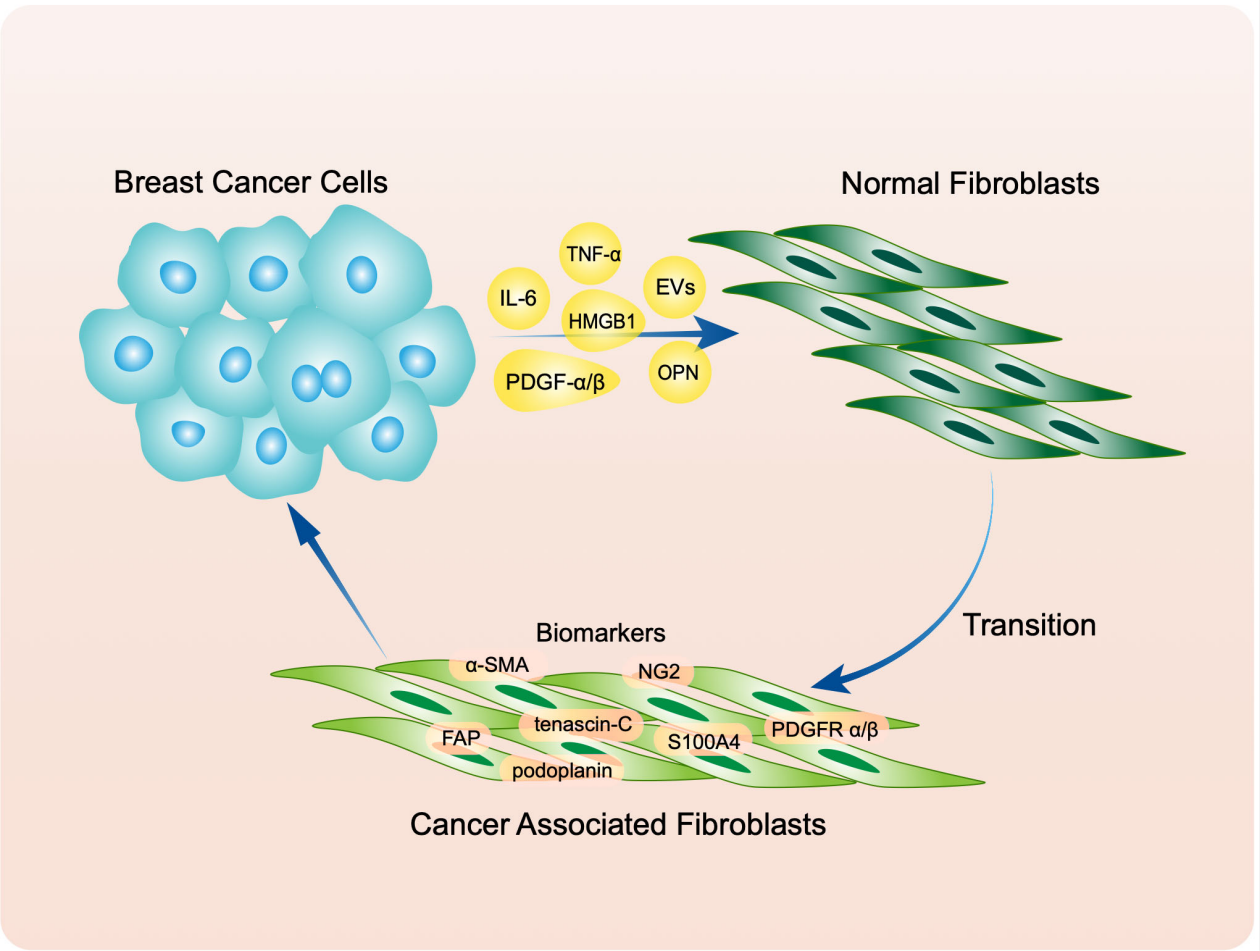
Breast cancer transfer normal fibroblasts (NFs) to cancer associated fibroblasts (CAFs) through secreting molecules including IL-6, TNF-α, PDGF α/β, OPN, HMGB1 and extracellular vesicles (EVs). Activated CAFs express classic biomarkers like α-SMA, tenascin-C, NG2, S100A4, PDGFR α/β, FAP and podoplanin.

NFs are typical tumor suppressor cells (54), but they are turned into “friends” by tumor cells to assist proliferation, migration, and invasion. Much evidence indicates that this transition is induced by secretion of cytokines by tumor cells. Some cytokines, including IL-6, basic fibroblast growth factor and PDGF-α/β, activate NFs through paracrine effects (55–59). In addition, the miR-9/EFEMP1 axis is crucial for activation (60). Another study demonstrated that as well as IL-6, breast cancer secretes TNF-α to stimulate KDM2A expression in normal mammary fibroblasts and transform them into CAFs (61). Butti et al. reported that tumor-derived osteopontin (OPN) engages CD44 and αvβ3 integrins on the fibroblast surface to induce myofibroblast differentiation and CXCL12 expression (62). These cytokines also take part in tumor metabolism. For instance, HMGB1 secreted by breast cancer cells promotes fibroblast activation via RAGE/aerobic glycolysis, and activated fibroblasts enhance breast cancer cell metastasis through increased lactate levels (63). Cancer cells also produce extracellular vesicles (EVs) that participate in this transition. Molecules including miR-370-3p, miR-125b, and miR-9 are carried by EVs to facilitate activation of NFs (64–66). The effects of cancer-cell-derived micro vesicles on fibroblast activation are regulated by the physical properties of the microenvironment (67). A joint medical-industrial study showed that pre-metastatic breast cancer cells could align ECM fibrils in a force-dependent manner, thereby allowing tumor-derived exosomes to reach the stroma more easily and convert NFs to CAFs (68) (**Figure 1**).

Mishra observed that both *in vitro* and *in vivo*, long-term co-culture of breast cancer cell condition medium and hMSCs led to differentiation of hMSCs into a myofibroblast phenotype, with upregulation of α-SMA, vimentin, fibroblast surface protein, and stromal derived factor 1 (SDF-1) (43). Another study showed that tumor-derived OPN transferred hMSC-to-CAF though the OPN-MZF1-TGF-β1 pathway (44). Moreover, Strong reported that co-culture of breast cancer cells and obese adipose derived stem cells (obASCs) also led to an increase in CAF biomarkers of obASCs (69). In tumors, bone mesenchymal stem cells (BMSCs) are recruited to tumor sites, resulting in their conversion into CAFs that aid tumor growth (70).

#### 2.3.2 Other pathways

As well as breast tumor cells, normal breast epithelial cells can induce the transition. De Vincenzo reported that c-Myc-expressing mammary epithelial cells could mobilize and activate NFs through the IGFs/IGF-IR axis, thereby establishing an environment for malignant transformation (71). Previous studies have reported that deletion of certain tumor suppressor genes in NFs, such as p53, p21, Pten and caveolin-1 (Cav-1), could activate oncogenic effects (72–75). Cav-1 downregulation may play a critical part in maintaining the aberrant status of breast-cancer-associated fibroblasts (76). Subsequently, a study reported that downregulation of p53 could transform NFs to CAFs, in a manner dependent on c-Ski-induced upregulation of SDF-1 (77). In low-stiffness stroma, loss of SPIN90-mediated microtubule acetylation was involved in CAF activation, which was associated with breast cancer progression (78).

Epithelial and endothelial cells become CAFs via epithelial–mesenchymal transition (EMT) and endothelial–mesenchymal transition, respectively (24, 79–81). A study showed that FOXF2-deficient breast cancer epithelial cells adopted a CAF-like phenotype (82). These cells are more likely to migrate to visceral organs by increasing autocrine TGF-β expression and enhancing aggressiveness of neighboring cells through increased paracrine TGF-β expression (82). The differentiation of CAFs gradually increases during tumor progression and may depend on the combined stimulation of TGF-β and SDF-1/CXCR4 autocrine signaling loops in CAFs to maintain stable differentiation (83, 84). TGF-β also plays this part through autophagy under oxidative stress in the TME (85, 86). Exhaustion of USP27X could inhibit TGFβ-induced fibroblast activation (87).

OPN could facilitate the transformation of mesenchymal stromal cells into CAFs, as well as increasing levels of CAF markers such as α-SMA, CXCL12, FSP-1, and tenascin-c specifically at tumor metastatic sites (88). Overexpression of miR-222 or knockdown of the LBR gene is enough to induce NFs to show CAF characteristics such as enhanced migration, invasion, and senescence; furthermore, conditioned medium from these cells increased migration and invasion of breast cancer cells (89). Chronic inflammation can induce the conversion of BMSCs to CAFs, leading to the production of pro-tumor inflammatory CAFs (90), a subtype of CAFs.

## 3 Subtypes of breast-CAFs

As mentioned above, CAFs are not a single cell type but comprise many subtypes, possibly owing to their various origins. Different methods are used to identify CAFs in breast cancer TME, results in different subtypes according to variety biomarkers (**Tables 1**, **2**). The subtypes of breast-CAFs, are supposed to play the same role in promoting breast cancer aggression via distinguished pathways, however, several CAFs can bring benefit for breast cancer treatment.

**Table 1:** Breast-CAFs identified by IHC or flow cytometry.

Subtypes	Biomarkers					Function
Brechbuhl HM et al. (91, 92)						
CD146 associated CAFs	CD146 positive					Reversal of tamoxifen resistance in ER positive breast cancer (91)
	CD146 negative					Enhance the drug resistance to tamoxifen (91) Promoted cancer metastasis and indicate poor prognosis of breast cancer patients (92)
Costa A et al. (93, 94)						
	FAP	CD29	αSMA	PDPN	PDGFRβ	
CAF-S1	High	Med-High	High	High	High	Play an important role in the immunosuppressive environment of breast cancer (93) Stimulate cancer cell migration and initiate EMT through CXCL12 and TGF-β pathways (94)
CAF-S2	Neg	Low	Neg-Low	Low	Low	Unknown
CAF-S3	Neg-Low	Med	Neg-Low	Low	Low-Med	Unknown
CAF-S4	Low-Med	High	High	Low	Med	Induce cancer cell invasion in three dimensions via NOTCH signaling (94)

**Table 2:** Breast-CAFs identified by single-cell sequencing.

Subtypes	DEGs/SDE genes	GO sets	Origin	Markers	Functions
Wu SZ et al. (95)					
**myCAFs**	MMP2/MMP11/FN1/LOX/PDPN/FAP/COL1A1/COL8A1/ COL11A1/COL12A1	Collagen biosynthesis and ECM regulatory pathways	Unknown	ACTA2/FAP/PDPN/COL1A1/COL1A2	Elevated capabilities for collagen secretion and alignment
**iCAFs**	IGF1/FIGF/CXCL12/DLK1/ CXCL13/CXCL1/IGF2/PDGFD/ ALDH1A1/ID2/EGFR/FGF10	Developmental signaling pathways and chemotactic regulation	Unknown	CXCL12	Associated with cytotoxic T-lymphocyte dysfunction
Bartoschek et al. (96)					
**vCAFs**	**Vascular regulators:** Notch3/Epas1/Col18a1/Nr2f2	Vascular development and angiogenesis	Perivascular location	Nidogen-2	Associated with blood vessels in early stages of tumor development
**mCAFs**	**Glycoproteins:** Dcn/Lum/Vcan **Structural proteins:** Col14a1 **Matricellular proteins:** Fbln1/Fbln2/Smoc **Matrix-modifying enzymes:** Lox/Loxl1 **Immune-cell-attracting factor:** CXCL14	Related to the ECM and EMT	Descendants of resident fibroblasts	Fibulin-1/ PDGFRα	Regulation of the tumor immune response
**cCAFs**	**Vascular regulators:** Notch3/Epas1/Col18a1/Nr2f2 **Cell cycle genes:** Nuf2/Mki67/Ccna2/Top2a/Cep55	Related to the cell cycle	Proliferating segment of vCAFs	Unknown	Represent the proliferative segment of vCAFs
**dCAFs**	**Various stem cell types:** Scrg1/Sox9/Sox10	Connected to differentiation of cells and development of tissues	Tumor cells that have undergone EMT	SCRG1	Tissue development

DEGs, differentially expressed genes; SDE, significantly differentially expressed; GO, gene ontology.

### 3.1 Identified by immunohistochemical (IHC) staining

In an earlier publication, breast CAFs were described as two types by IHC staining, depending on whether they were positive or negative for CD146, and this description was used to evaluate their role in estrogen receptor (ER)-dependent proliferation and tamoxifen sensitivity (91). The results showed that tamoxifen sensitivity of tamoxifen-resistant breast cancer cells could be restored after co-culture with CD146-positive CAFs. On the other hand, CD146 negativity was correlated with inferior clinical response to tamoxifen and worse patient outcomes (91). Recently, the same research team reported that CD146-negative CAFs could promoted tumor metastasis and predict the possibility of lymph node metastasis in small primary tumors (92). These findings provide an experimental basis for clinical precision therapy.

### 3.2 Identified by multicolor flow cytometry

Mechta-Grigoriou et al. characterized four CAF subsets, CAF-S1, S2, S3, and S4, using multicolor flow cytometry (fluorescence-activated cell sorting) and found that the CAF-S1 subset had a key role in the immunosuppressive milieu of breast cancer (93). Two years later, they confirmed the presence of these four subpopulations in metastatic lymph nodes and described their biomarkers and functions (94).

### 3.3 Identified by single-cell sequencing

Single-cell sequencing has been a hot topic in recent years. Wu SZ el al. used this technique to divide CAFs into myofibroblast-like CAFs (myCAFs) and inflammatory CAFs (iCAFs) in TNBC (95). The biomarkers of myCAFs are ACTA2, FAP, PDPN, COL1A1, and COL1A2; and CXCL12 (SDF-1) is a biomarker of iCAFs (95). Another study showed that iCAFs were from CD26 positive NFs and myCAFs were from CD26 negative NFs (97). Bartoschek et al. also used it to distinguish four subtypes of breast-cancer-associated fibroblasts: vascular CAFs (vCAFs), matrix CAFs (mCAFs), developmental CAFs (dCAFs), and cycling CAFs (cCAFs) (96). The terms are relatively clear and easy to understand. This description is more detailed than previous ones and includes origin, significantly differentially expressed genes, gene ontology (GO) sets, markers, and functions. The team validated expression profiles in clinical samples (96). Experiments *in vitro* showed that both vCAFs and mCAFs could promote tumor invasion and may represent potential targets for clinical therapies (96). Sebastian et al. also used single-cell sequencing to identify six CAF subsets in BALB/c-derived 4T1 mammary tumors with distinct gene expression profiles (98).

## 4 Functions of CAFs in breast cancer metastasis

As mentioned in the introduction, cancer metastasis relies on multiple steps, including growth at the primary site, EMT, invasion into the systemic circulation, dissemination via circulation, extravasation into the target organ, and finally growth at the metastatic site (5). CAFs participate in all these steps. Most previous reviews have summarized the roles of CAFs at different stages. Our review, however, focuses on the ways in which CAFs function and the results achieved.

### 4.1 Secret molecules to assist cancer metastasis

**Figure 2:**
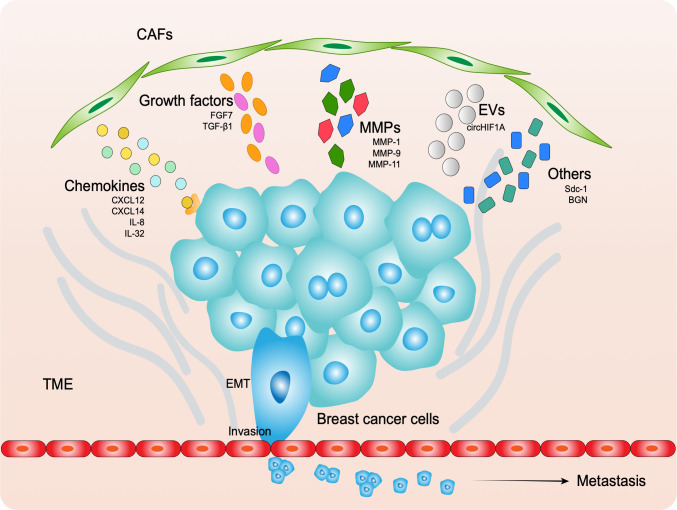
CAFs secret molecules to assist cancer EMT, invasion and metastasis, including chemokines, growth factors, MMPs, EVs and others.

Many of studies have demonstrated that CAFs assist the progression of breast cancer cells though paracrine signaling by various molecules, including biological macromolecules, cytokines, and enzymes, as well as exosomes (99) (**Figure 2**).

#### 4.1.1 Chemokines

SDF-1, currently known as CXCL12, directly binds to its receptor CXCR4, a G-protein-coupled receptor, to induce tumor angiogenesis, thereby promoting breast cancer growth (100). OPN-driven CAFs release CXCL12 to initiate EMT in tumor cells (62). CAF-enriched primary tumor stroma can mimic the CXCL12-rich bone metastatic niche and can thus be used to help identify potentially metastatic cancer cells (101). CXCL12 stimulates the proliferation of CD44-positive and CD24-negative breast cancer stem cells (102). The diabetes drug metformin can prevent the production of CXCL12 and IL-8 by CAFs, which is associated with increased phospho-AMP kinase levels. Therefore, metformin can be used to interrupt HIF-1α-driven SDF-1 signaling in CAFs to decrease breast cancer invasion (103). The same family member CXCL14 can promote tumor growth by stimulating angiogenesis and recruiting macrophages through nitric oxide synthase 1 (NOS1) (104). CXCL14-induced NOS1 or ACKR2 downregulation attenuates EMT and migration (105). IL-32, also known as NK4, secreted by CAFs stimulates the invasion and metastatic potential of breast cancer cells via activation of integrin β3-p38 MAPK (106). IL-6 produced by CAFs stimulates the signal transducer and activator of transcription 3 (STAT3) pathway, promoting breast cancer cell growth and radioresistance (107).

#### 4.1.2 Growth factors

Growth factors are known to have significant effects on mammary cells. Studies have shown that breast stromal fibroblasts produce FGF7, which has profound effects on epithelial and myoepithelial cells. For instance, Palmieri et al. used immunohistochemistry to demonstrate FGF7 expression in stroma of lobular carcinomas and invasive ductal carcinomas, and illustrated with Matrigel-embedded organoids that FGF7 increases cell proliferation (108). By secreting another growth factor, TGF-β1, CAFs activate the TGF-β/Smad signaling pathway in breast cancer cells, promoting their aggressive phenotype, which involves enhanced cell–ECM adhesion, migration, invasion, and EMT (109).

#### 4.1.3 MMPs

There are 26 human MMPs, which can be classified into six groups according to their substrate specificity and homology. During tumor invasion and metastasis, MMPs are essential for the degradation of stromal connective tissue and basement membrane components. During ECM remodeling, MMPs contribute to tumor progression primarily by degrading ECM (110). Studies have shown that CAF-derived MMP-1 and MMP-9 promote the invasion of breast cancer cells (111, 112). Another study showed that MMP-11 (stromelysin-3) was preferentially expressed in the stroma of tumors and was associated with poor prognosis (113–115).

#### 4.1.4 EVs

EVs are membrane-bound vesicles released into the extracellular microenvironment by both prokaryotic and eukaryotic cells. They are composed of several lipid-bilayer nanosized vesicles. In a recent study, Luga et al. (116) reported that CAFs secrete exosomes that activate Wnt-planar cell polarity signaling in breast cancer cells, promoting cell motility and metastasis. Moreover, in breast cancer stem cells, hypoxic CAFs release exosomes to transfer circHIF1A, which regulates miR-580-5p by sponging CD44 expression (117).

#### 4.1.5 Others

CAF-derived Sdc1 is associated with significantly higher micro-vascular density and larger vessel area, which stimulate breast carcinoma growth and angiogenesis (118). Furthermore, another secretory protein, biglycan (BGN) was found to be upregulated in CAFs compared with normal cancer-adjacent fibroblasts. Notably, BGN expression was negatively correlated with CD8+ T cells and was associated with poor prognoses, possibly because of the immunosuppressive TME (119).

### 4.2 Functions through other molecules

Some molecules are not secreted by CAFs but interact with them to promote the progression of breast cancer. Some regulate CAFs to assist cancer metastasis, whereas others are affected by CAFs. Gene regulation techniques can be used to investigate the function of CAFs.

Protein-kinase-R-like endoplasmic reticulum kinase (PERK), which is selectively activated by Rho-associated kinase (ROCK) in mammary tumor epithelium, recruits and persists cancer-promoting CAFs (120). A CAF phenotype is generated by myeloid zinc finger-1 phosphorylation in mesenchymal stem cells, which leads to an increase in the stemness of cancer cells (121). Autophagy and survival are enhanced by Nox4 and Nrf2 pathway activation in CAFs during tumorigenesis and metastasis of breast cancers (122). In a mixed xenograft and indirect co-culture model, estrogen was shown to induce CAFs to activate FGF2/FGFR1 paracrine signaling and trigger expression of connective tissue growth factor (CTGF), leading to migration and invasion of breast cancer cells (123). Conversely, CAF-conditioned media induced ER ubiquitination and proteasomal degradation of MCF7 and T47D cells (124). The activation of PI3K-AKT signaling by C3a-C3aR enhances pro-metastatic cytokine secretion and expression of ECM components by CAFs. In mouse models of breast cancer, treating mice with genetic or pharmacological inhibitors of C3aR signaling effectively inhibits lung metastasis (125). It has been shown that Gremlin1 (GREM1), which has high expression in CAFs, abrogates bone morphogenetic protein (BMP)/SMAD signaling and promotes the mesenchymal phenotype, stemness, and invasion of breast cancer cells, which are associated with poor prognosis regardless of molecular subtype (126). Phosphodiesterase 5 (PDE5) is highly expressed in CAFs and also contributes to the progression of breast cancer and affects clinical outcomes (127). Furthermore, CAFs enhanced GPNMB expression in breast cancer cells in an organotypic model of tumor-stroma interactions (128); however, its mechanism is unclear. According to Soon et al. (129), CAFs induce significantly more EMT molecular markers in MCF7 cells than NFs, as manifested by increased vimentin expression, whereas E-cadherin expression was decreased in MCF7 cells.

The role of some molecules in CAF can be better understood through gene regulation techniques. Overexpression of mitochondrial uncoupling proteins (UCP-1) in CAFs can induce mitochondrial dysfunction by enhancing β-oxidation to produce ketone bodies and vesicles enriched with ATP, acting as fuel for tumor growth (130). Knockout of adhesion kinase (FAK) in CAFs did not affect primary tumor growth and proliferation but significantly limited breast cancer metastasis via exosomal microRNA-mediated intercellular communication (131). In addition, STAT1 depletion in CAFs reduced periductal reactive fibrosis and retarded the progression of early breast cancer *in vivo*, suggesting that STAT1 contributes to tumorigenesis from the stroma (132).

### 4.4 Function as a bodyguard for breast cancer cells

#### 4.4.1 Co-metastasis with cancer cells

When tumors invade blood vessels or lymphatic vessels, CAFs form clusters with cancer cells. The CAFs protect the cancer cells from immune attack, enable them to endure fluid mechanical force, and reduce their apoptosis, as well as improving vessel invasion. Metastatic lung cancer cells that co-metastasized with their own CAFs from the primary site were found to grow more rapidly than those that did not in a tumor metastasis mouse model (133). Alternatively, removing cancer-associated factors results in a significant reduction in metastatic cancer cells. Cancer cells and CAFs extravasate through blood vessels or lymphatic vessels, resulting in metastatic lesions in the appropriate organs. In the new environment, CAFs can survive and continuously secrete growth factors and cytokines to stimulate the growth of metastatic cancer cells. These findings warrant further investigation into which subgroups of CAFs co-metastasize with tumor cells and to find biomarkers that distinguish them and may provide new targets for tumor therapy.

#### 4.4.2 Suppressing immunity

There is evidence that tumors with CAF-rich microenvironments exhibit immunologically cold environments, suggesting that therapeutically targeting a specific CAF subpopulation in breast could improve clinical outcomes (134). In addition, an animal study demonstrated that CAFs impaired the function of tumor-infiltrated immune cells *in vivo* and significantly promoted breast tumor progression (135). The IL6-adenosine positive feedback loop is mediated by CD73+ gamma delta regulatory T cells (Tregs), which further promote IL6 secretion by CAFs via adenosine/A2BR/p38MAPK signaling. The infiltration of CD73+ gamma delta Tregs also impaired the tumoricidal activities of CD8+ T cells, and this effect was associated with significantly worse patient prognosis. It appears that IL6-adenosine loops between CD73+ gamma delta Tregs and CAFs play a critical part in promoting tumor progression and immunosuppression in breast cancer (136). Timperi E et al. suggests that lipid-associated macrophages (LAM) recruited via the CAF-driven CXCL12-CXCR4 axis support an immunosuppressive microenvironment by acquiring protumorigenic functions (137).

#### 4.4.3 Assisting tumor metabolism

In the TME, stromal cells cooperate with cancer cells metabolically. Tumor cells can utilize CAF metabolic byproducts to feed anabolic metabolism and proliferation, indicating that metabolic symbiosis has an important role in tumor growth (138). Some studies have helped to elucidate the metabolic dynamics between cancer cells and CAFs. For example, FATP1, which is relatively highly expressed in CAFs, has been proposed as a potential target for disrupting breast cancer cell lipid transfer (139). Another study showed that extracellular ATP promotes interactions between breast cancer cells and fibroblasts, where S100A4 is produced in collaboration with breast cancer cells to exacerbate breast cancer metastasis (140). Aspartate derived from CAFs sustains cancer cell proliferation, whereas glutamate derived from CAFs promotes remodeling of the ECM (141).

### 4.5 Other findings

The authors recently proposed a novel tumor invasion mechanism based on invasive cancer cells migrating independently on elongated CAFs (CAF fibers) embedded in a three-dimensional collagen matrix. This mechanism involves cancer cells interacting with fibronectin fibrils assembled on CAFs primarily through integrin-α5β1 (142). A study by Gao et al. (143) demonstrated that CAFs located within human breast cancer interface zones have a significant role in inducing EMT. The same results can be obtained by artificially altering the expression of certain molecules in CAFs. In cancer tissue, mechanical pressure is also believed to play a part in the mechanisms (144, 145). In cancer cells expressing the matrix-remodeling CAF receptor Endo180 (MRC2), genetic deletion profoundly limits tumor growth and metastasis (146). MDA-MB-231 cells are accelerated (1) by direct physical interactions, where activated fibroblasts penetrate the matrix and act as scaffolds for coalescence and aggregation; and (2) through release of soluble accelerating factors such as MMPs or, in the case of activated normal human dermal fibroblasts (NHDFs), SDF-1α/CXCL12 (147).

## 5 Clinical value of CAFs in breast cancer

CAFs have an important role in the development of breast cancer and are involved in tumor cell occurrence, growth, angiogenesis, cell invasion, and metastasis. Some of these molecular markers can provide a data basis for the determination of breast cancer types, the choice of treatment, and prediction of patient prognosis. CAFs secrete products that regulate tumor cells and have a positive impact on clinical outcomes. Therefore, the clinical application value of CAFs for breast cancer has attracted the attention of many researchers worldwide.

### 5.1 Relationship of CAF-associated molecules with clinical diagnosis and prognosis

CAF-associated molecules are related to the clinical outcomes of breast tumors and metastasis, and survival of patients. They are important indicators of prognosis and can enable early intervention for breast cancer patients, as well as helping to provide new strategies (148).

#### 5.1.1 CAF biomarkers

After α-SMA was stained in 60 invasive breast cancer patients, computer-aided image analysis showed that the expression of α-SMA significantly differed between the metastasis group and the non-metastasis group. The metastasis group showed high α-SMA expression and a significantly lower overall survival rate (149). α-SMA can cause cancer cell metastasis and reduce overall survival, because α-SMA-positive CAFs can promote tumor growth through OPN secretion (150).

PDGF receptor expression is associated with unfavorable prognosis in breast cancer when the PDGF pathway is dysregulated. High PDGFRα expression has been linked to aggressive subtypes of breast cancer including TNBC, and high PDGF-CC expression increases the risk of 5-year distant recurrence. Moreover, PDGFR expression in tumor cells has been reported to be significantly elevated in lymph node metastases and asynchronous recurrences (151). Another study used double immunostaining of α11 integrin and PDGFRβ in human breast cancer samples and associated normal tissues of DCIS patients (152). The results showed that invasive ductal cancer (IDC) had higher densities of integrin-11 or PDGFR than DCIS tumors, which suggested that integrin α11 is mainly expressed by a subset of PDGFRβ-positive CAFs in human breast cancer. Furthermore, patient outcomes were analyzed with respect to the integrin α11/PDGFRβ+ CAF subgroup, showing that an increase in stromal density of integrin α11/PDGFR was associated with higher tumor grade, metastasis, and patient mortality. Recently, Akanda, M. R. et al. reported that in breast cancer brain metastasis patients, expression of PDGFR-β in the stroma of metastasis site was associated with recurrence free survival (153).

In addition, FAP expressed by CAFs is an independent factor predicting the prognosis of breast cancer patients, and the expression of FAP is correlated with cancer cell metastasis and survival (154). An IHC study of 449 patients with DCIS who had undergone extensive resection and did not receive radiotherapy and chemotherapy concluded that FAP-α and GOLPH3 overexpression were highly specific for the recurrence and progression of DCIS and may thus represent novel tumor markers for progression of DCIS to invasive breast cancer (IDC) (155). In breast tumors with a high stromal content, radiolabeled FAP-specific enzyme inhibitor (FAPIs) may offer high contrast for fast imaging and could serve as anti-tumor agents (156). In 68Ga-FAPI positron emission tomography/computed tomography, remarkably high uptake and image contrast were observed for several widely prevalent cancers. These findings could lead to new applications such as noninvasive tumor identification, staging examinations, or the use of radioligand therapy (157).

#### 5.1.2 MMPs

Immunohistochemical analysis of 154 breast cancer patients and 42 women without tumor disease revealed that postmenopausal patients, hormone-receptor-positive patients, and histological ductal carcinoma patients had higher MMP-1 staining intensity and higher MMP3 staining percentages and intensities (158). A clinical study of 48 patients with breast cancer and 13 patients with benign breast disease found that expression levels of MMP-9 mRNA were significantly increased in breast cancer patients compared with patients with benign breast disease. In addition, plasma MMP-2 and MMP-9 were significantly reduced in breast cancer patients after surgery, so both MMP-2 and MMP-9 could be used as markers of breast cancer disease response to therapy (159). On the other hand, MMP-2 expression was correlated with tumor size and neovascularization, MMP-9 expression was correlated with hormone receptor status, and stromal cell co-expression of MMP-2 and MMP-9 was significantly associated with tumor size. Therefore, these markers could be used in combination to assess the prognosis of breast cancer patients (160). MMP-9, MMP-11, and TIMP-2 expression by CAFs were also significantly associated with poor prognosis in luminal A tumors (161).

#### 5.1.3 CAV-1

CAV-1 is a structural protein involved in the trafficking of vesicles and signaling in caveolae, which are sphingolipid-rich invaginations of the plasma membrane. Cell lines and primary breast cancers from humans contain negative CAV-1 RNA and protein levels, and CAV-1 reintroduced *in vitro* inhibits many tumorigenic properties, including anchorage independent growth (162). A study analyzed 669 tumor specimens with TNBC by immunohistochemistry, showing that lack of stromal CAV-1 expression in TNBC was significantly associated with worse overall survival; conversely, increased mRNA levels of CAV-1 in 141 tumor samples were associated with better overall survival (163). Another study showed that low expression of CAV-1 in breast cancer stroma was associated with early recurrence, progression, tamoxifen resistance, and 5-year survival, especially in invasive micropapillary carcinoma, whereas CAV-1 gene expression promoted EGFR signal transduction, which mediates tyrosine kinase activity and was found to be an effective marker for breast cancer diagnosis and prognosis (164).

#### 5.1.4 Others

It has been demonstrated that tumor grade is significantly related to a high level of immunostaining for AUF1 in both cancer cells and adjacent CAFs (165). Locally advanced breast cancer patients with high levels of DNMT1 in breast stromal fibroblasts have poor survival, because DNMT1 promotes angiogenesis through IL-8/VEGF-A upregulation (166). In TNBC patients, increased CAF activation was linked to increased infiltration of polarized CD163-positive tumor-associated macrophages (TAMs), as well as lymph node metastasis. CAF activation, TAM infiltration, and lymph node metastasis were shown to be independent prognostic factors for disease-free survival in TNBC patients (167). Din contrast to factors with high expression, depletion of FAK in CAFs prompts its activation of protein kinase A via CCR1/CCR2 on cancer cells, leading to increased glycolysis in malignant cells, which mediates the metabolism of malignant cells, reduces overall patient survival, and leads to poor prognosis of breast cancer patients (168). There has been evidence that the five-gene prognostic CAF signature (RIN2, THBS1, IL1R1, RAB31, and COL11A1) is not only effective for predicting prognosis, but also for estimating clinical immunotherapy response (169).

To sum up, substances related to CAFs, transformation of CAFs by deletion of molecular markers, and derivatives of CAFs and tumor suppressor genes have clinical applications in the diagnosis, prognosis, and treatment of breast cancer. They can also be used to provide data and information for precision treatment of breast cancer patients and speed up the process of breast cancer treatment.

### 5.2 Relationship of CAF-associated molecules with treatment

As mentioned above, CAFs and related molecules have an important impact on the prognosis of breast cancer and can thus be explored as targets for the treatment of breast cancer. Treatment plans for breast cancer include vaccines, reducing drug resistance, and mediating the radiation resistance of CAFs.

#### 5.2.1 Anti-FAP vaccines

FAP is one of the most important biomarkers of CAFs and is primarily expressed on the CAF surface. Many anti-tumor therapies focus on FAP. It is possible to increase the potency of T-cell-mediated anti-tumor effects by targeting FAPα, and combination of a dual-targeting vaccine with doxorubicin effectively increased the anti-tumor activity of the vaccine by decreasing immunosuppressive factors and promoting the infiltration of tumor cells by lymphocytes; this finding may provide useful guidance for clinical research on the combination of DNA vaccination with low-dose chemotherapy (170). Another chemotherapy drug, cyclophosphamide, together with FAPα-targeted modified vaccinia Ankara, have been shown to be effective in overcoming immunosuppression and improving specific anti-tumor immune responses (171). Importantly, mice vaccinated with FAP and given cyclophosphamide chemotherapy showed significant tumor growth suppression (inhibition ratio: 80%) and longer survival times (172). Another vaccine reduced the growth of 4T1 tumors by promoting production of cytotoxic T lymphocytes that killed CAFs, and the decrease in FAPα-expressing CAFs markedly decreased collagen I and other stromal factors, resulting in a marked attenuation of tumor progression (173). Another study developed a tumor vaccine prepared from tumor-cell-derived exosome-like nanovesicles (eNVs-FAP), which demonstrated excellent anti-tumor effects in a variety of tumor-bearing mouse models. According to mechanistic analysis, eNVs-FAP stimulated dendritic cell maturation, increased infiltration of effector T cells into tumor cells and FAP+ CAFs and decreased the number of immunosuppressive cells such as M2-like TAMs, myeloid-derived suppressor cells, and Tregs in the TME. In addition, FAP+ CAF clearance enhanced ferroptosis via interferon-gamma (174). Another drug delivery agent, functionalized nanocaged HFn-FAP, could specifically enhance targeted therapy for CAFs when administered intravenously in TNBC (175).

#### 5.2.2 Reversal of drug resistance

Antitumor drug resistance is among the main culprits in breast cancer recurrence and metastasis. Recent studies on drug resistance involving CAFs have mainly focused on tamoxifen, anti-HER2 drugs, and chemotherapy. Drug resistance to breast cancer can be reversed through treatments that reduce CAF activity, thereby reducing recurrence and metastasis. Tamoxifen is an ER modulator used for endocrine therapy in patients with ER-positive breast cancer. According to a study, tamoxifen resistance in breast cancer may be caused by CD63+ CAFs via exosomal miR-22 (176). CAFs with upregulation of HMGB1 expression and secretion via GPR30/PI3K/AKT signaling enhanced MCF-7 cell resistance to TAM by increasing autophagy dependent on ERK activity (177). The TAF/FGF5/FGFR2/c-Src/HER2 axis is responsible for HER2-targeted therapy resistance in breast cancer, which can be reversed by FGFR inhibitors (178). In patients with HER2-positive breast cancer, CAF-derived NRG1 contributes to trastuzumab resistance through high expression of HER3/AKT, but combination with pertuzumab may reverse resistance (179). Cancer-derived xenografts engraft successfully when CD10+GPR77+ CAFs are present, and treating these CAFs with an anti-GPR77 antibody eliminates tumor formation and restores tumor chemosensitivity (180). Claudin-low TNBCs are resistant to chemotherapy when CAF activates IFN signaling. Inhibition of this pathway could improve breast cancer outcomes in a novel way (181). A study showed that gMG treatment significantly retards tumor growth, reduces CAF production, and improves DOX sensitivity in a DOX-resistant TNBC tumoroid-bearing mouse model, because it was found that INFG/STAT1/NOTCH3 is a molecular link between breast cancer stem cells and CAFs, and it’s expression was increased in DOX-resistant TNBC cell lines, as well as CAF-transformation and self-renewal ability (182).

#### 5.2.3 Reversal of radiation resistance

CAFs have been shown to be radioresistant and to undergo significant changes in oxidative metabolism indices. It is likely that CAFs that survive radiation treatment influence the fate of associated cancer cells. Identifying these CAFs, determining their mode of communication with cancer cells, and eradicating them, especially when they exist at the margins of a radiotherapy target volume, may improve cancer treatment effectiveness (183). Dendrigraft poly-l-lysine (DGL)/gemcitabine (GEM)@PP/GA nanoparticles for TAF-targeted regulation and deep tumor penetration have been reported. When MMP-2 was overexpressed in the TME, GEM-conjugated small nanoparticles (DGL/GEM) are released from DGL/GEM@PP/GA, causing the large nanoparticles (PP/GA) loaded with 18beta-glycyrrhetinic acid (GA) to accumulate at the tumor site. The released DGL/GEM can penetrate deep into the tumor to release GEM intracellularly and kill tumor cells. It is also possible that residual GA-loaded nanoparticles may accumulate around tumor vessels and be absorbed more efficiently by TAFs, which regulate the secretion of Wnt16, an essential damage response program (DRP) molecule, around tumor vessels. When DGL/GEM@PP/GA was applied to breast cancer models with stroma-rich stroma, significant and long-term anti-tumor effects were observed (184).

## 6 Conclusions and prospecs

CAFs, the most abundant cells in breast cancer stroma, secrete various ECM components, growth factors, cytokines, proteins, enzymes, and hormones. CAFs participate in the development and progression of breast cancer by stimulating epithelial cell malignant transformation, tumor initiation, tumor growth, ECM degradation, tumor angiogenesis, and cancer cell invasion and metastasis. Furthermore, CAFs are valuable in the clinical diagnosis of breast cancer, as well as in therapy and prediction of prognosis. However, many aspects remain unclear, including the relationship between CAFs and other mesenchymal cells or stroma structures, such as tunneling nanotubes, which are made by exosomes derived from breast cancer cells but not CAFs (185), the precise mechanism of their escape from immune attack, and whether other valuable molecular markers of CAFs exist. The breast cancer immune-microenvironment with tumor-associated tertiary lymphoid structure (TA-TLS) usually have a higher representation of tumor-infiltrating lymphocytes (TIL) and show a better response to chemotherapy and immunotherapy (186). A study showed that TA-TLS could be coordinated by FAP^neg^ CAFs that exhibit characteristics like lymphoid tissue organizers and their roles are to facilitate anti-tumor immunity and immune response to checkpoint immunotherapy (187). Unfortunately, this study was done in melanoma, not breast cancer. In conclusion, the role of CAFs in breast cancer warrant further investigation.

## Author contributions

YL conceptualized the manuscript, YL and CW did literature search and wrote it. TH designed the figures. XY and BT critically reviewed it. All authors contributed to the article and approved the submitted version.
